# How Individual and Situational Factors Influence Measures of Affective and Cognitive Theory of Mind in Psychiatric Inpatients

**DOI:** 10.3389/fpsyg.2022.855038

**Published:** 2022-05-19

**Authors:** Magdalena Knopp, Juliane Burghardt, Bernhard Meyer, Manuel Sprung

**Affiliations:** ^1^Division of Clinical Psychology, Department of Psychology and Psychodynamics, Karl Landsteiner University of Health Sciences, Krems an der Donau, Austria; ^2^Department of Psychology, Faculty of Psychology and Educational Sciences, Ludwig-Maximilians-Universität München, Munich, Germany; ^3^Psychiatric Rehabilitation Clinic Gars am Kamp, Psychosomatisches Zentrum Waldviertel, Gars am Kamp, Austria; ^4^Psychosomatisches Zentrum Waldviertel, University Hospital for Psychosomatic Medicine Eggenburg, Eggenburg, Austria

**Keywords:** cognitive empathy, mentalizing, personality disorder, substance abuse, gender differences, age difference

## Abstract

Mental disorders are associated with difficulties to correctly infer the mental states of other’s (theory of mind; ToM). These inferences either relate to affective states of others (affective ToM) or to their thoughts, intentions, or beliefs (cognitive ToM) and can be associated with mental disorder. The current study explores the influence of individual and situational effects on the measurement of ToM abilities within two clinical samples, to increase generalizability. We analyzed data from 229 in-patients; 103 patients treated for alcohol use disorder and 126 patients treated for a personality disorder. ToM was assessed with the Movie for the Assessment of Social Cognition (MASC). We analyzed changes in test performance over the course of the test using a logistic linear mixed effects model. Performance on the cognitive ToM items decreased over time, while performance on the affective ToM items increased over time. This difference was more pronounced among older individuals. The results show important moderators of ToM performance that might help to resolve inconsistencies in the current literature about ToM abilities in different clinical or age groups.

## Introduction

Many mental disorders are associated with difficulties to correctly infer the mental states of others, i.e., theory of mind (Németh et al., 2018). ToM deficits are seen in over 30 mental disorders (Cotter et al., 2018). These inferences either relate to affective states of others (affective ToM) or to their thoughts, intentions, or beliefs (cognitive ToM). To promote the understanding of ToM and its association with mental disorders, the influence of individual, and situational factors on the assessment of ToM needs to be considered.

Two meta-analyses and a systematic review provide evidence for ToM impairment in individuals with alcohol use disorder (AUD) relative to healthy controls (HC; Onuoha et al., 2016; Bora and Zorlu, 2017; Sanvicente-Vieira et al., 2017). Onuoha et al. (2016) summarized the result from 8 studies in their meta-analysis and found that AUD individuals (*N* = 187) displayed reduced ToM compared to HC (*N* = 187), with a large effect size, Hedges’ g = –1.62 [95% CI = –2.28; 0.96]. A second meta-analysis summarizing the results from 12 studies reported that AUD individuals (*N* = 317) compared to HC individuals (*N* = 298) displayed ToM deficits with a medium effect size, Cohen’s d = 0.58 [95% CI = 0,36–0.81] (Bora and Zorlu, 2017). Furthermore, ToM impairment is common among borderline personality disorder (BPD) patients (Németh et al., 2018) and has also been demonstrated in patients with other personality disorders, for instance patients with narcissistic personality disorder or cluster C personality disorders (Ritter et al., 2011; Marissen et al., 2012; Herpertz and Bertsch, 2014). However, only few studies have compared ToM abilities in patients with BPD and other personality disorders. One such study found no difference between BPD and other personality disorders in overall ToM performance, but rather ToM impairment was associated with the severity of psychopathology (Normann-Eide et al., 2020). Deficits in affective and cognitive ToM can show different associations with mental disorders. For instance, a study by Harari et al. (2010) found that patients with BPD only have cognitive ToM deficits, but later studies showed deficits in affective and cognitive ToM (Petersen et al., 2016). In line with this, a meta-analysis concluded that BPD have difficulties with cognitive as well as affective ToM tasks (Németh et al., 2018). In contrast to patients with BPD, individuals with AUD show deficits primarily in affective ToM, but their cognitive ToM is comparable to healthy controls (HC) (Maurage et al., 2016). Thus, BPD and AUD differ regarding cognitive and affective ToM abilities. Considering this dissociation, findings about ToM deficits should not be generalized to other mental disorders without further testing. Thus, affective and cognitive ToM abilities are linked to mental health. Further, they can be influenced by other characteristics of the individual. For instance, gender or age can be differentially associated with affective and cognitive ToM abilities. Women show more pronounced affective responses (Han et al., 2008) and perform better in tasks that assess affective ToM (Baron-Cohen, 2010). Further, they often show better emotion recognition (Hall and Matsumoto, 2004; Hoffmann et al., 2010; Donges et al., 2012). Many studies have found a decrease in ToM abilities in older adults, which was summarized in a meta-analysis by Henry et al. (2013). This meta-analysis found reliable aging effects on both affective and cognitive ToM. However, some recent studies found effects of aging were limited to the cognitive ToM and did not occur for the affective ToM (Wang and Su, 2013). A similar study found that age was associated with a decrease in cognitive ToM but not in affective ToM (Bottiroli et al., 2016).

Besides differentiating between affective and cognitive ToM, ToM deficits can further be divided into different error types: exceeding ToM, less ToM, no ToM. Multiple studies found that individuals with BPD showed an exceeding ToM, this means that patients with BPD displayed a tendency to interpret incidental actions in an intentional way (Sharp and Vanwoerden, 2015; Normann-Eide et al., 2020). In contrast, a meta-analysis by Onuoha et al. (2016) concluded that individuals with AUD typically displayed reduced ToM compared to healthy controls.

The present study aims to explore the influence of individual and situational effects on the measurement of affective and cognitive ToM abilities in two clinical samples, patients from an AUD treatment unit and patients from a personality disorder (PD) treatment unit; with a focus on mixed personality disorder and borderline personality disorder. The two AUD and PD samples are very different in their sociodemographic characteristics, especially regarding age, and gender and also show different ToM errors and a distinct pattern of affective and cognitive ToM. In order to detect general influences of individual and situational factors on the measurement of affective and cognitive ToM abilities using data from two distinct samples can increase the generalizability of results. Since the patients with PD in the present sample are younger and more often women, we assume that they will outperform individuals with AUD, who are older and more often men, on affective and cognitive ToM. Differential effects of age, gender, and test duration on affective and cognitive ToM performance are investigated. We expect a decrease in task performance over the course of the test. Based on previous studies we assume that age, gender, and test duration will influence ToM performance. Further we expect women and younger participants to outperform men and older participants. These effects of age, gender, and test duration may be different for affective and cognitive ToM.

## Materials and Methods

### Participants and Procedure

We collected data from 390 patients from an inpatient psychiatric-psychosomatic clinic in Austria, which focusses on patients with chronic mentally illnesses (216 AUD, 174 PD). The inpatient stays last between 2 and 3 months, during which the patients receive intense therapy by an interdisciplinary team. Respective data was collected as part of the routine examination at admission.

Only data from outcome assessment at the time of intake were included in the analyses. To be admitted to the clinic patients need to be able to undergo therapies. This means they need to have at least conversational skills in German, show no marked cognitive deficits and show the motivation to engage in therapy. Exclusion criteria are acute psychosis, danger of suicide and intoxication (Knopp et al., 2021). Patients were either in a treatment unit for AUD or for PD (i.e., mixed personality disorder and borderline personality disorder). All AUD patients were recently detoxified. Data from 75 patients (64 AUD, 11 PD) were excluded due to missing values. To control for the possible influence of overall test compliance we excluded data from 86 participants who answered less than 4 out of 6 MASC control items correctly (49 AUD, 37 PD). We also analyzed the data without excluding participants who failed more than 2 out of 6 MASC control questions; this did not alter results in any significant way. After exclusion, the final AUD sample included 103 patients (66 men) with a mean age of 49.0 (*SD* = 8.9) years. The final PD sample included 126 patients (33 men) with a mean age of 35.0 (*SD* = 11.2) years. Sociodemographic variables are depicted in Table 1. Most patients were native speakers of German (AUD: 95, PD: 118).

**TABLE 1:** Sociodemographic-, illness-related characteristic and MASC test results (%) *.

Sociodemographic characteristics	PD *N* (126)	AUD *N* (103*)*	χ^2^ (df)	*p*
Sex (men/women)	26.2/73.8	64.1/35.9	41.1 (3)	**<0.001**
Educational level Low/medium/high	71.5/19.8/8.7	68.1/20.3/11.6	146.5 (5)	**<0.001**
Employment (yes/no)	17.7/82.3	42.2/57.8	61.0 (3)	**<0.001**
**Illness-related characteristics**				
Duration of sick leave <3/3–6/ > 6 months	49.5/4.6/45.9	68.2/4.5/27.3	96.4 (5)	**<0.001**
Duration of symptoms <3/3–6/ > 6 months	6.4/1.6/92	14.6/6.8/78.6	301.7 (5)	**<0.001**
**MASC**	***M (SD)***	***M (SD)***	***t* (df)**	***p***
ToM total (%)	69.2 (13.3)	63.8 (14.2)	-2.9 (212)	**0.004**
ToM exceeding	15.8 (7.0)	16.1 (7.7)	0.4 (210)	0.705
ToM less	13.9 (6.8)	16.7 (7.9)	2.9 (202)	**0.004**
ToM no	6.8 (5.2)	8.0 (5.0)	1.7 (222)	0.098
Affective ToM	70.1 (15.4)	63.4 (16.9)	–3.1 (209)	**0.002**
Cognitive ToM	68.5 (15.8)	64.0 (16.6)	2.1 (213)	**0.038**
Control questions	80.7 (12.4)	79.9 (12.2)	–0.5 (219)	0.645

Bold p-values are p < 0.005. *Percentage without missing.

In the AUD sample, the primary diagnosis of 101 patients was alcohol dependence syndrome (F10.2); 2 patients were diagnosed with harmful use of alcohol (F10.1). Additionally, patients in the AUD sample had a multitude of comorbid (secondary) diagnoses: 33 affective disorders (F31-38), 9 anxiety disorders (F40-42), 6 personality disorders (F60-61), 2 posttraumatic stress disorders (F43.1), 1 eating disorder (F50), 2 obesity (E66). In the PD sample, the primary diagnoses were: 51 mixed personality disorder (F61) and 46 borderline personality disorder (F60.3), 9 patients were diagnosed with other personality disorders (F60, F68), 7 with posttraumatic stress disorder and other reactions to severe stress (F43), 5 recurrent depressive disorder (F33), 5 phobic and other anxiety disorders (F40-42), 2 with bipolar affective disorder (F31) and 1 with Asperger syndrome (F84.5).

### Procedure

The data for this study were collected between July 2017 and May 2019 as part of the routine outcome monitoring. Basic sociodemographic data, such as age and sex, were obtained from the hospital information system. All other measures were collected using the Computer-based Health Evaluation System (Holzner et al., 2012). The admission examination spans two 1-h sessions with self-report questionnaires and the ToM measure. Patients answer the questions in a computer assessment room with eight separated cubicles; so up to eight patients complete the questionnaires and the ToM task in one assessment session.

### Measures

ToM was assessed using a behavioral measure. Sociodemographic variables were assessed by self-report.

#### Movie for the Assessment of Social Cognition

ToM was measured with the Movie for the Assessment of Social Cognition (MASC), which relies on video sequences of people interacting with each other to assess cognitive and affective ToM performance (Dziobek et al., 2006). The MASC shows a series of video clips of social interactions, which concern friendship and dating themes. The format is highly immersive. After each video clip participants are required to infer the thoughts or feelings of the individual(s) in the particular scene. Participants respond to multiple choice (MC) questions, with four possible answers. These MC-questions include one correct and three incorrect answers. Incorrect answers represent three prototypical error types. These errors types are: (a) “exceeding ToM,” i.e., a mental state is attributed when there is no mental explanation for the situation, (b) “less ToM,” i.e., a present mental state is misattributed, and (c) “no ToM,” i.e., a total absence of mental inference (e.g., making attributions of physical causality to social situations and mental states). Test items either require inferences about a characters’ feelings/emotions (affective ToM) or their thoughts/intentions (cognitive ToM). The stimulus set includes more cognitive than affective ToM items, therefore the raw scores were transformed into “percentage correct.” Additionally, in order to control variables such as memory, general compliance and understanding of the task, six control questions that make no reference to mental states are included in the MASC. The control questions require inferences about non-social/non-mental state related situations or facts and are also in a MC-format. The MASC has previously been reported to have high reliability; internal consistency was high (α ≥ 0.82 for all scores), good test-retest stability and good convergent and discriminant validity (Fossati et al., 2018). The average duration to complete the MASC was 30.5 min.

#### Sociodemographic Characteristics

We assessed sex, age, education level, employment, duration of sick leave, duration of symptoms, and duration of psychotherapy. Sex was measured dichotomously; “0” for women and “1” for men. Age ranged from 18 to 74 years and entered regressions as a continuous variable. For the visualization of results, we used age groups (18–34; 35–45; 46–53; 54–75) based on quartiles. Educational level was coded into three graduation levels; “1” low education level (compulsory school), “2” medium education level (middle school), and “3” high education level (high school or university degree). Employment status was coded into employed (fulltime or part time) vs. unemployed (retired, unemployed, early retirement). Duration of sick leave was coded “1” if duration of sick leave has been present less than 3 months, “2” if duration of sick leave has been present 3–6 months and “3” if duration of sick leave has been present for more than 6 months. Duration of symptoms was coded “1” if symptoms have been present less than 6 months, “2” if symptoms have been present 6–12 months and “3” if symptoms have been present for more than 12 months. Duration symptoms was measured using a single item (“How long ago did you first perceive the problems that led to the treatment?”).

### Data Analysis

Comparative statistics (Student’s *t*-test or Pearson’s Chi-squared test) for both patient groups (AUD vs. PD) were calculated, to determine their complementary clinical characteristics and to replicate previous reports of specific ToM performance patterns.

Within-subject effects were analyzed with an item-level analysis, utilizing a Logistic Linear Mixed Effects model (LME), with individual MASC item responses as dichotomous (correct vs. incorrect) dependent measure. The analysis used two LME models to test effects of test duration, age, and ToM facet on ToM performance. Both LME models included the main effects of item order (marker of test duration), ToM facet (affective vs. cognitive), age, and sex, and the interaction of item order and ToM facet. (1) The first model additionally contained the interaction effects of ToM facet with sex, item order with sex and the three-way interaction of item order, ToM facet and sex.

(2) The second model instead included interaction effects of ToM facet with age, item order with age, and the three-way interaction of ToM facet, item order, and age. Resulting *p*-values for all interaction effects within the two regression models were corrected for multiple testing using the Bonferroni method (7 tests in total: p_*adjusted*_ = 0.007). Analyses were performed using R 4.0.2^1^ with the packages lme4 and lmerTest for LME modeling (glmer, family: binomial), tidyverse, magrittr, dplyr, and reshape2 for data preparation, and ggplot2 for data visualization.

## Results

### Sociodemographic and Illness-Related Characteristic

Table 1 depicts the sociodemographic characteristic, duration of symptoms/illness, and treatment history. The ratio of men and women differed between the AUD and the PD samples. There were more men than women in the AUD sample. There were more women than men in the PD sample. In both samples the majority of patients was unemployed. The proportion of unemployed individuals was higher in the PD sample. In line with this, patients in the PD sample had a lower education level than patients in the AUD sample. The majority of both samples reported a low education level (AUD participants 68.1%; PD 71.5%). The proportion of patients with low education level was even higher in the PD sample.

Regarding the history of symptoms/illness, patients in the PD sample more frequently reported a longer period of sick leave (duration of sick leave) and a longer history of subjectively experienced symptoms (duration of symptoms) than AUD patients. Moreover, 71.0% of patients in the PD sample reported being in psychotherapy (prior to the current inpatient treatment) for more than a year, compared to 46.6% of the participants in the AUD sample.

### Movie for the Assessment of Social Cognition Test Results

We report the results after excluding participants that failed more than 2 of MASC control questions, however, the results are highly similar with the full sample. The MASC results for the two samples are presented in Table 1. On average patients in the AUD sample answered 79.9% (*SD* = 12.2) of the MASC control items correctly, while in the PD sample answered 80.7% (*SD* = 12.4) of the control items correctly. Regarding ToM, items patients in the AUD sample answered *M* = 63.8 (*SD* = 14.2) of the MASC items correctly and patients in the PD sample answered *M* = 69.2 (*SD* = 13.3) of the MASC items correctly. The PD sample scored higher on the affective ToM M = 70.1 (*SD* = 15.4) compared to the AUD sample *M* = 63.4 (*SD* = 16.9), *t* (209) = –3.1, *p* = 0.002. Furthermore, the performance in the cognitive ToM facet of patients in the PD sample, *M* = 68.5 (*SD* = 15.8), was better than of patients in the AUD sample, *M* = 64.0 (*SD* = 16.6), *t* (213) = 2.1, *p* = 0.038. This difference was evident for both affective and cognitive ToM, although the difference was more pronounced for affective ToM (see Table 1). The most common error type among patients in the AUD sample was “less ToM,” *M* = 16.7, *SD* = 7.9, which reflects misattribution of a mental state. This error type was significantly less frequent among patients in the PD sample, *t*(202) = 2.9, *p* = 0.004. Among patients in the PD “exceeding ToM” was the most common error types *M* = 15.8 (*SD* = 7), however, it was equally common among patients in the AUD sample.

**FIGURE 1:**
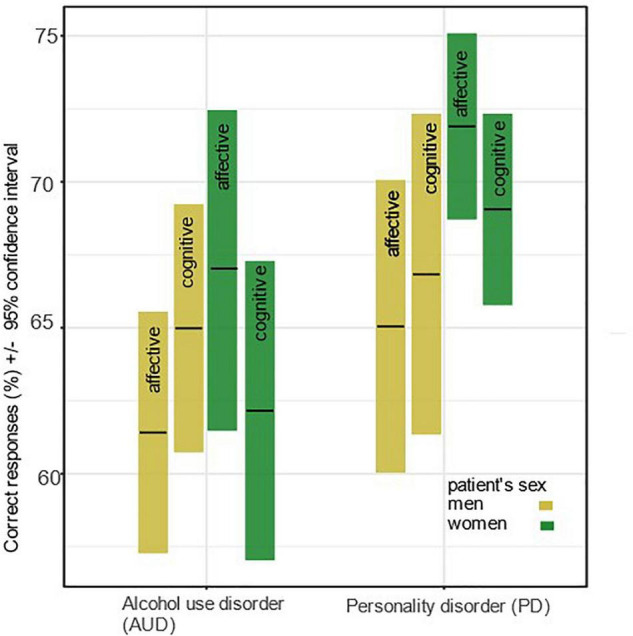
Mean correct responses (%) and 95% confidence intervals for ToM performance by ToM facet, disorder, and participant’s sex.

Table 2 and Figure 1 show the results of the first regression model, which tested main and interaction effects of item order (of MASC test items), the two ToM facets (affective vs. cognitive), and of participant’s age and sex on ToM performance, and all interactions with sex. The analysis revealed that MASC performance decreased over the duration of the test, β = 0.18, *SE* = 0.06, *p* = 0.003. This effect was mainly due to a decrease in cognitive ToM performance. In contrast, affective ToM showed a positive slope and thus increased over time. Female participants outperformed male participants in their overall ToM performance, β = 0.29, *SE* = 0.11, *p* = 0.010, however, this effect was no longer significant after a Bonferroni correction was applied, which resulted in a critical *p*-value of *p* = 0.007. However, the interaction between ToM facet and participant sex was significant (β = –0.32, *SE* = 0.10, *p* = 0.002), suggesting that women outperformed man on the affective ToM, while their performance was comparable or lower on the cognitive ToM facet.

**TABLE 2:** Regression of factors influencing ToM performance.

	β	*SE*	*z*	*p*
ToM facet (cognitive vs. affective)	0.08	0.08	1.10	0.288
Item order (z-values)	0.18	0.06	3.00	**0.003**
Sex_*patient*_ (woman = 0, man = 1)	0.29	0.11	2.60	0.010
Age (z-values in years)	–0.19	0.05	–4.00	**<0.001**
ToM facet × item order	–0.31	0.08	–4.00	**<0.001**
ToM facet × sex_*patient*_	–0.32	0.10	–3.10	**0.002**
Item order × sex_*patient*_	0.06	0.08	0.70	0.471
ToM facet × item order × sex_*patient*_	–0.04	0.10	–0.30	0.732

*Bold p-values are p < 0.005.*

**FIGURE 2:**
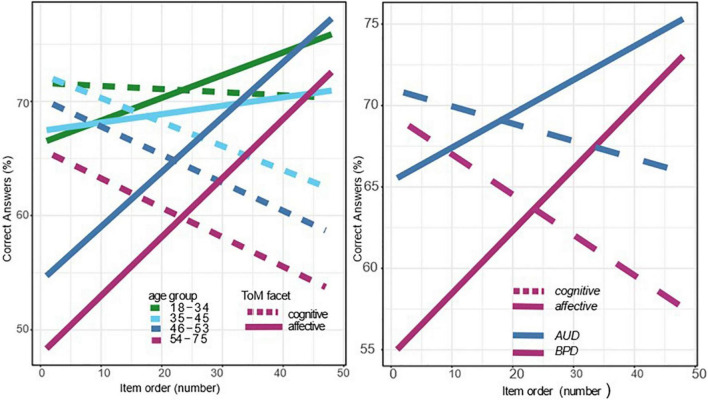
Course of affective and cognitive ToM performance over the test duration.

The second regression model tested main and interaction effects of item order, ToM facet (affective vs. cognitive), and participant sex (male vs. female) and all interactions with age. Results show a significant main effect of participant sex. These effects are visible in Figure 2, on the left side. There was also a significant interaction of the ToM facet with item order, β = –0.31, *SE* = 0.08, *p* < 0.001. Moreover, the decline in cognitive ToM was more pronounced for older patients, as indicated by a significant three-way interaction of ToM facet with item order and age, β = –0.15, *SE* = 0.05, *p* = 0.004. Figure 2 illustrates this effect, showing that the slopes of the MASC test performance over the course of the testing are more pronounced among older patients. To visualize these results Figure 2 shows four age groups. However, there was also significant main effect of age, suggesting an overall decrease in ToM with increasing age, β = –0.19, *SE* = 0.05, *p* < 0.001. The chart on the right side of Figure 2 shows the ToM performance separately for the two samples (AUD vs. PD) and for the two ToM facets (affective vs. cognitive ToM).

## Discussion

The results support the notion that performance in ToM tasks, specifically the MASC, is influenced by individual and situational factors. ToM performance decreased during the course of the test. However, this progression differed between affective and cognitive ToM. While cognitive ToM decreased, affective ToM increased during the course of the test. These decreases could have resulted from a decline in task motivation, attention, or fatigue among participants. Previous studies have shown that cognitive ToM relies more strongly on working memory and attention (Gabriel et al., 2019) than affective ToM. Hence, affective ToM might be less influenced by a decrease in attention.

The pattern of decreasing cognitive ToM and increasing affective ToM was more pronounced among older participants than younger adults. Thus, the results are more in line with the studies that have shown a negative effect of aging on the cognitive ToM only (Wang and Su, 2013; Bottiroli et al., 2016) instead to those that found effects of aging on both cognitive and affective ToM to be equally strong (Henry et al., 2013). The decline in cognitive ToM performance in older adults has previously been associated with a decrease in executive functioning (Wang and Su, 2013) and a strategic reallocation of scarce cognitive resources. Therefore the motivation to engage in cognitively demanding activities is reduced (Hess, 2014). This might explain why the affective ToM shows an increase in performance over the course of the test. Participants might choose to allocate resources to attend to emotions of actors within the MASC. The results are in line with earlier studies that showed that increased task motivation enhanced older adults’ performance in such a way their ToM performance level reached younger adults’ level of ToM performance Zhang et al. (2018). In line with this, our findings show strong performance differences at the end of the test, when motivation is presumably low. Based on these effects it is interesting to note that in the MASC all interactions are presented by relatively young actors. Their young age might decrease the emotional closeness between the participants and the actors (Hess, 2014; Zhang et al., 2018). As a result, this could lead to lower task performance among older participants.

Further, the results showed that while women outperform men on the affective ToM, men outperform women on the cognitive ToM. This is in line with previous findings showing a higher emotional competence in women as marked by their better performance on tasks measuring the affective dimension of social cognition. Especially women showed a better decoding of non-verbal messages (Hall et al., 2000), and as well as a higher ability to recognize emotions from facial expressions (Hall and Matsumoto, 2004; Hoffmann et al., 2010; Donges et al., 2012).

There were significant differences in the overall ToM performance between the two patient samples. AUD patients had more difficulties inferring mental states than PD patients. However, despite the differences in the samples regarding their symptomatology, socio-demographic characteristics, and distinct ToM errors and pattern of affective and cognitive ToM the two samples displayed similar effects over the course of the test, which suggests that the influence of individual and situational factors may be general.

### Limitation

The current study did not directly measure general cognitive abilities, executive functioning, or task motivation. This limits our understanding of the processes that underly the findings, future studies should assess motivation directly to test its effect. However, to reduce the possible influence of general cognitive abilities, executive function, or task engagement, we excluded data from participants who failed more than 2 out of 6 of MASC control questions. Thus, the results in the MASC test questions are likely to reflect genuine ToM abilities and not merely general cognitive abilities.

Since the data was collected within the standard treatment routine it was not possible to counterbalance item order. It is theoretically possible that the items in later parts of the task would be more difficult than in the beginning, which would be an alternative explanation for the effect of item order However, attributing the entire effect of item order to differences in item difficulty is complicated by the age effects. It might be possible that item difficulty increases for cognitive items and decreases for affective items. However, assuming that item difficulty increases specially for older individuals and only on cognitive items is relatively unlikely. Therefore, we assume that differences in item difficulty are not a suitable explanation for effects of item order.

Even though the two samples have a higher generalizability than a single sample, the findings are nevertheless limited and should be replicated with a broader spectrum of mental disorders.

### Conclusion

The present study demonstrates how strongly performance in ToM tasks can be influenced by individual factors, i.e., sex, age, and disorder type (AUD vs. PD). The effects of age and sex should be kept in mind, when comparing results between different clinical samples, as many clinical samples have a typical distribution of age, and sexes. Further, the results illustrate the influence of situational factors, i.e., test duration, on ToM performance, since the test performance changed over the course of the test. This effect was different for affective and cognitive ToM. Cognitive ToM decreased over time, while affective ToM increased over time. Thus, the influence of individual and situational factors may explain the heterogenous evidence of ToM deficits in different mental disorders. Moreover, considering the influence of individual and situational factors can improve the validity of assessment of ToM performance.

## Data Availability Statement

The datasets presented in this article are not readily available because of the vulnerability of the study sample. Participants of this study did not agree for their data to be shared publicly, so supporting data are not available. Requests to access the datasets should be directed to MK, magdalena.knopp@kl.ac.at.

## Ethics Statement

The studies involving human participants were reviewed and approved by the Ethics Commission of the Karl Landsteiner University of Health Sciences (Nr: 1020/2021). The patients/participants provided their written informed consent to participate in this study.

## Author Contributions

MK, JB, and MS: conceptualization. MK, JB, and BM: methodology, formal analysis, investigation, and data curation. MK and JB: writing—original draft preparation. MS: writing—review and editing. All authors have read and agreed to the published version of the manuscript.

## Conflict of Interest

The authors declare that the research was conducted in the absence of any commercial or financial relationships that could be construed as a potential conflict of interest.

## Publisher’s Note

All claims expressed in this article are solely those of the authors and do not necessarily represent those of their affiliated organizations, or those of the publisher, the editors and the reviewers. Any product that may be evaluated in this article, or claim that may be made by its manufacturer, is not guaranteed or endorsed by the publisher.

## Footnotes

1
http://cran-r-project.org/

## References

[B1] Baron-Cohen S. (2010). Empathizing, systemizing, and the extreme male brain theory of autism. *Prog. Brain Res.* 186 167–175. 10.1016/B978-0-444-53630-3.00011-7 21094892

[B2] Bora E. Zorlu N. (2017). Social cognition in alcohol use disorder: a meta-analysis. *Addiction* 112 40–48. 10.1111/add.13486 27287050

[B3] Bottiroli S. Cavallini E. Ceccato I. Vecchi T. Lecce S. (2016). Theory of Mind in aging: comparing cognitive and affective components in the faux pas test. *Arch. Gerontol. Geriatr.* 62 152–162. 10.1016/j.archger.2015.09.009 26434925

[B4] Cotter J. Granger K. Backx R. Hobbs M. Looi C. Y. Barnett J. H. (2018). Social cognitive dysfunction as a clinical marker: a systematic review of meta-analyses across 30 clinical conditions. *Neurosci. Biobehav. Rev.* 84 92–99. 10.1016/j.neubiorev.2017.11.014 29175518

[B5] Donges U.-S. Kersting A. Suslow T. (2012). Women’s greater ability to perceive happy facial emotion automatically: gender differences in affective priming. *PLoS One* 7:e41745. 10.1371/journal.pone.0041745 22844519 PMC3402412

[B6] Dziobek I. Fleck S. Kalbe E. Rogers K. Hassenstab J. Brand M. (2006). Introducing MASC: a movie for the assessment of social cognition. *J. Autism Dev. Disord.* 36 623–636. 10.1007/s10803-006-0107-0 16755332

[B7] Fossati A. Borroni S. Dziobek I. Fonagy P. Somma A. (2018). Thinking about assessment: further evidence of the validity of the Movie for the Assessment of Social Cognition as a measure of mentalistic abilities. *Psychoanal. Psychol.* 35 127–141. 10.1037/pap0000130

[B8] Gabriel E. T. Oberger R. Schmoeger M. Deckert M. Vockh S. Auff E. (2019). Cognitive and affective Theory of Mind in adolescence: developmental aspects and associated neuropsychological variables. *Psychol. Res.* 85 533–553. 10.1007/s00426-019-01263-6 31701225 PMC7900042

[B9] Hall J. A. Carter J. D. Horgan T. G. (2000). “Gender differences in nonverbal communication of emotion,” in *Gender and Emotion: Social Psychological Perspectives*, ed. Fischer A. H. (Cambridge: Cambridge University Press), 97–117. 10.1017/CBO9780511628191.006

[B10] Hall J. A. Matsumoto D. (2004). Gender differences in judgments of multiple emotions from facial expressions. *Emotion* 4 201–206. 10.1037/1528-3542.4.2.201 15222856

[B11] Han S. Fan Y. Mao L. (2008). Gender difference in empathy for pain: an electrophysiological investigation. *Brain Res.* 1196 85–93. 10.1016/j.brainres.2007.12.062 18221733

[B12] Harari H. Shamay-Tsoory S. G. Ravid M. Levkovitz Y. (2010). Double dissociation between cognitive and affective empathy in borderline personality disorder. *Psychiatry Res.* 175 277–279. 10.1016/j.psychres.2009.03.002 20045198

[B13] Henry J. D. Phillips L. H. Ruffman T. Bailey P. E. (2013). A meta-analytic review of age differences in theory of mind. *Psychol. Aging* 28 826–839. 10.1037/a0030677 23276217

[B14] Herpertz S. C. Bertsch K. (2014). The social-cognitive basis of personality disorders. *Curr. Opin. Psychiatry* 27 73–77. 10.1097/YCO.0000000000000026 24270477

[B15] Hess T. M. (2014). Selective engagement of cognitive resources: motivational influences on older adults’ cognitive functioning. *Perspect. Psychol. Sci.* 9 388–407. 10.1177/1745691614527465 26173272 PMC5911399

[B16] Hoffmann H. Kessler H. Eppel T. Rukavina S. Traue H. (2010). Expression intensity, gender and facial emotion recognition: WOmen recognize only subtle facial emotions better than men. *Acta Psychol.* 135 278–283. 10.1016/j.actpsy.2010.07.012 20728864

[B17] Holzner B. Giesinger J. M. Pinggera J. Zugal S. Schöpf F. Oberguggenberger A. S. (2012). The Computer-based Health Evaluation Software (CHES): a software for electronic patient-reported outcome monitoring. *BMC Med. Inform. Decis. Mak.* 12:126. 10.1186/1472-6947-12-126 23140270 PMC3529695

[B18] Knopp M. Riffer F. Burghardt J. Sprung M. (2021). Geschlechtsspezifische Unterschiede in der psychotherapeutischen Versorgung. *Psychotherapeut* 66 511–517. 10.1007/s00278-021-00523-4

[B19] Marissen M. A. E. Deen M. L. Franken I. H. A. (2012). Disturbed emotion recognition in patients with narcissistic personality disorder. *Psychiatry Res.* 198 269–273. 10.1016/j.psychres.2011.12.042 22406389

[B20] Maurage P. D’Hondt F. de Timary P. Mary C. Franck N. Peyroux E. (2016). Dissociating affective and cognitive theory of mind in recently detoxified alcohol-dependent individuals. *Alcoholism* 40 1926–1934. 10.1111/acer.13155 27427391

[B21] Németh N. Mátrai P. Hegyi P. Czéh B. Czopf L. Hussain A. (2018). Theory of mind disturbances in borderline personality disorder: a meta-analysis. *Psychiatry Res.* 270 143–153. 10.1016/j.psychres.2018.08.049 30248485

[B22] Normann-Eide E. Antonsen B. T. Kvarstein E. H. Pedersen G. Vaskinn A. Wilberg T. (2020). Are impairments in theory of mind specific to borderline personality disorder? *J. Pers. Disord.* 34 827–841. 10.1521/pedi_2019_33_417 30785865

[B23] Onuoha R. C. Quintana D. S. Lyvers M. Guastella A. J. (2016). A meta-analysis of theory of mind in alcohol use disorders. *Alcohol Alcohol.* 51 410–415. 10.1093/alcalc/agv137 26755641

[B24] Petersen R. Brakoulias V. Langdon R. (2016). An experimental investigation of mentalization ability in borderline personality disorder. *Compr. Psychiatry* 64 12–21. 10.1016/j.comppsych.2015.10.004 26608042

[B25] Ritter K. Dziobek I. Preißler S. Rüter A. Vater A. Fydrich T. (2011). Lack of empathy in patients with narcissistic personality disorder. *Psychiatry Res.* 187 241–247. 10.1016/j.psychres.2010.09.013 21055831

[B26] Sanvicente-Vieira B. Romani-Sponchiado A. Kluwe-Schiavon B. Brietzke E. Araujo R. B. Grassi-Oliveira R. (2017). Theory of mind in substance users: a systematic minireview. *Subst. Use Misuse* 52 127–133. 10.1080/10826084.2016.1212890 27617349

[B27] Sharp C. Vanwoerden S. (2015). Hypermentalizing in borderline personality disorder: a model and data. *J. Infant Child Adolesc. Psychother.* 14 33–45. 10.1080/15289168.2015.1004890

[B28] Wang Z. Su Y. (2013). Age-related differences in the performance of theory of mind in older adults: a dissociation of cognitive and affective components. *Psychol. Aging* 28 284–291. 10.1037/a0030876 23276219

[B29] Zhang X. Lecce S. Ceccato I. Cavallini E. Zhang L. Chen T. (2018). Plasticity in older adults’ theory of mind performance: the impact of motivation. *Aging Ment. Health* 22 1592–1599. 10.1080/13607863.2017.1376313 28885057

