# Corrigendum: How individual and situational factors influence measures of affective and cognitive theory of mind in psychiatric inpatients

**DOI:** 10.3389/fpsyg.2024.1488243

**Published:** 2024-09-26

**Authors:** Magdalena Knopp, Juliane Burghardt, Bernhard Meyer, Manuel Sprung

**Affiliations:** ^1^Division of Clinical Psychology, Department of Psychology and Psychodynamics, Karl Landsteiner University of Health Sciences, Krems an der Donau, Austria; ^2^Department of Psychology, Faculty of Psychology and Educational Sciences, Ludwig-Maximilians-Universität München, Munich, Germany; ^3^Psychiatric Rehabilitation Clinic Gars am Kamp, Psychosomatisches Zentrum Waldviertel, Gars am Kamp, Austria; ^4^Psychosomatisches Zentrum Waldviertel, University Hospital for Psychosomatic Medicine Eggenburg, Eggenburg, Austria

**Keywords:** cognitive empathy, mentalizing, personality disorder, substance abuse, gender differences, age difference

In the published article, there was an error in the author list, and author Friedrich Riffer was erroneously included. The corrected author list appears below:

Magdalena Knopp, Juliane Burghardt, Bernhard Meyer and Manuel Sprung

The updated Author contributions section appears below:

MK, JB, and MS: conceptualization. MK, JB, and BM: methodology, formal analysis, investigation, and data curation. MK and JB: writing—original draft preparation. MS: writing—review and editing. All authors have read and agreed to the published version of the manuscript.

The updated Acknowledgments section appears below:

We like to thank Isabel Dziobek for providing the Movie for the assessment of social cognition (MASC), and we also like to thank Isabel Dziobek, Pierre Maurage, and Ulrike Buhlmann for providing the MASC item numbers for the calculation of the affective and cognitive Theory of Mind subscales. We express our gratitude to Assoc. Prof. Prim. Dr. Friedrich Riffer for his contributions to the study.

The authors apologize for this error and state that this does not change the scientific conclusions of the article in any way. The original article has been updated.

